# Negative Regulation of the Novel *norpA^P24^* Suppressor, *diehard4*, in the Endo-lysosomal Trafficking Underlies Photoreceptor Cell Degeneration

**DOI:** 10.1371/journal.pgen.1003559

**Published:** 2013-06-06

**Authors:** Jongwoo Lee, Myungchul Song, Sujeong Hong

**Affiliations:** 1 Subtropical Horticulture Research Institute, Jeju National University, Jeju, Korea; University of Buffalo, United States of America

## Abstract

Rhodopsin has been used as a prototype system to investigate G protein-coupled receptor (GPCR) internalization and endocytic sorting mechanisms. Failure of rhodopsin recycling upon light activation results in various degenerative retinal diseases. Accumulation of internalized rhodopsin in late endosomes and the impairment of its lysosomal degradation are associated with unregulated cell death that occurs in dystrophies. However, the molecular basis of rhodopsin accumulation remains elusive. We found that the novel *norpA^P24^* suppressor, *diehard4*, is responsible for the inability of endo-lysosomal rhodopsin trafficking and retinal degeneration in *Drosophila* models of retinal dystrophies. We found that *diehard4* encodes *Osiris 21*. Loss of its function suppresses retinal degeneration in *norpA^P24^*, *rdgC^306^*, and *trp^1^*, but not in *rdgB^2^*, suggesting a common cause of photoreceptor death. In addition, the loss of *Osiris 21* function shifts the membrane balance between late endosomes and lysosomes as evidenced by smaller late endosomes and the proliferation of lysosomal compartments, thus facilitating the degradation of endocytosed rhodopsin. Our results demonstrate the existence of negative regulation in vesicular traffic between endosomes and lysosomes. We anticipate that the identification of additional components and an in-depth description of this specific molecular machinery will aid in therapeutic interventions of various retinal dystrophies and GPCR-related human diseases.

## Author Summary

Malfunctioning of phototransduction is the major cause of human blindness. Without functional phototransduction, rhodopsin-1, the major visual pigment, is rapidly endocytosed and accumulated in late endosomes. Impaired lysosomal delivery of endocytosed rhodopsin and its degradation has been reported to trigger progressive and light-dependent retinal degeneration in *Drosophila* models. It is intriguing why endocytosed rhodopsin accumulates in late endosomes instead of being delivered to lysosomes for degradation. Is this attributable to a saturation of rhodopsin endocytosis, which impedes the delivery capacity of the cell? To investigate the underlying mechanisms of rhodopsin accumulation in late endosomes, we used a suppressor of phototransduction mutants, which was identified previously from our unbiased genetic screen. This suppressor, called *diehard4*, shifts the membrane balance between late endosomes and lysosomes, resulting in the facilitated degradation of endocytosed rhodopsin. Our results clearly demonstrate that a previously unknown mechanism of negative regulation is actively engaged in vesicular traffic between endosomes and lysosomes in fly photoreceptors. We showed that eliminating such blockage alone was enough to rescue retinal degeneration in phototransduction mutants. From these results, we anticipate that the identification of additional components and an in-depth description of this molecular machinery will aid in therapeutic interventions of various retinal dystrophies and neurodegenerative disorders.

## Introduction

Retinitis pigmentosa is the most common form of retinal degeneration and the major cause of human blindness [1]. Most degenerative retinal dystrophies are caused by various genetic mutations. Malfunctioning of phototransduction is the predominant cause of retinal dystrophies, in that most genes involved in the functioning and regulation of the phototransduction cascade are directly or indirectly related to retinal degeneration syndromes [2], [3]. Therefore, it is not surprising that rhodopsin-1, the major visual pigment, was the first molecule identified as a target for such mutations [4], [5].

*Drosophila norpA* (phospholipase C, PLC) acts as a central effector molecule in phototransduction [6]. It has been used as an invertebrate model for studying molecular mechanisms of retinal degeneration caused by malfunctioning of the phototransduction cascade [7]. Interestingly, cGMP phosphodiesterase, which relays the signal from G-proteins in vertebrate phototransduction, is also known to trigger retinal degeneration in mouse models [8]–[10]. The loss of *norpA* function essentially shuts down the phototransduction cascade, resulting in a failure to raise intracellular Ca^2+^ levels through light-sensitive channels. Thus, Ca^2+^-dependent enzymes required for rhodopsin recycling cannot be activated, resulting in the formation of the stable rhodopsin-arrestin complex [11]–[14]. It has been reported that excessive endocytosis followed by the formation of stable rhodopsin-arrestin complexes and accumulation of internalized rhodopsin in late endosomes trigger apoptosis in *norpA* mutant photoreceptor cells [12].

The “granule group” genes in *Drosophila* have been known for their vital role in lysosomal biogenesis and functioning [15], [16]. A previous study found that the functional loss of the “granule group” genes resulted in rhodopsin accumulation in the Rab7-positive late endosomes and triggered retinal degeneration in *norpA* mutant photoreceptor cells [12], [17]. Therefore, the accumulation of internalized rhodopsin in late endosomes and impaired endo-lysosomal trafficking clearly causes retinal degeneration in both the *norpA* and the “granule group” mutant photoreceptors. However, the molecular basis of this pathologic accumulation remains unknown.

The role of excessive endocytosis of light-activated rhodopsin on saturating the capacity of the trafficking machinery for the endo-lysosomal progression, resulting in the accumulation of endocytosed rhodopsin in the late endosomes remains controversial. Alternatively, previously unknown regulatory mechanisms prevent endocytosed rhodopsin from further movement toward lysosome. A growing number of evidences support the fact that the eukaryotic cell utilizes active regulatory mechanisms in monitoring and maintaining the intracellular membrane balance of the endo-lysosomal system [18]–[21]. Therefore, it is imperative to identify genetic components underlying rhodopsin accumulation and present epistatic evidences that possibly override the endo-lysosomal blockage in phototransduction mutants.

Triplo-lethal (Tpl) locus, cytologically defined as the 83D4-E2 region in chromosome 3 in *Drosophila*, was identified as a sole locus responsible for both triplo-lethality and haplo-lethalith in segmental aneuploids [22]. Point mutations responsible for the Tpl phenotype remain unidentified [23], although the Ell product, a transcription elongation factor, was found to be a suppressor of the Tpl phenotype [24]. Therefore, it is proposed that this phenotype is caused by a gene cluster that shows at least partial redundancy and its dosage is critical to its function [25].

The Osiris gene family was identified in an effort to explain the Tpl phenotype as an effect of a gene cluster. This is a large conserved family, with most genes (20 of 23) located within the Tpl locus [26]. Although the cellular function of the Osiris family of proteins is currently unknown, all members share characteristic features, including endoplasmic reticulum signal sequences, a pair of cysteine residues near the amino terminus, a putative transmembrane domain, an AQXLAY motif, and a number of endocytic signaling motifs such as YXXØ [26], [27].

Previously, we screened for *norpA^P24^* suppressors by random mutagenesis. The screening had the advantage of the yeast site-specific recombination *FLP-FRT* system and could identify both essential and nonessential genes [28]. Here we report that the novel *norpA^P24^* suppressor, *diehard4* (*die4*), is responsible for the inability of endo-lysosomal rhodopsin trafficking and retinal degeneration in *norpA^P24^* mutants. We found that *die4* encodes *Osiris 21* (*Osi21*). A loss of function of *Osi21* suppresses retinal degeneration in various phototransduction mutants. In addition, the loss of function shifts the membrane balance between endosomes and lysosomes, resulting in the facilitated degradation of endocytosed rhodopsin. Our results demonstrate that the existence of negative regulation in vesicular traffic between endosomes and lysosomes. This mechanism may trigger retinal degeneration in phototransduction mutants.

## Results

### The Novel *norpA^P24^* Suppressor, *die4*, Encodes *Osiris 21*

**Figure 1:**
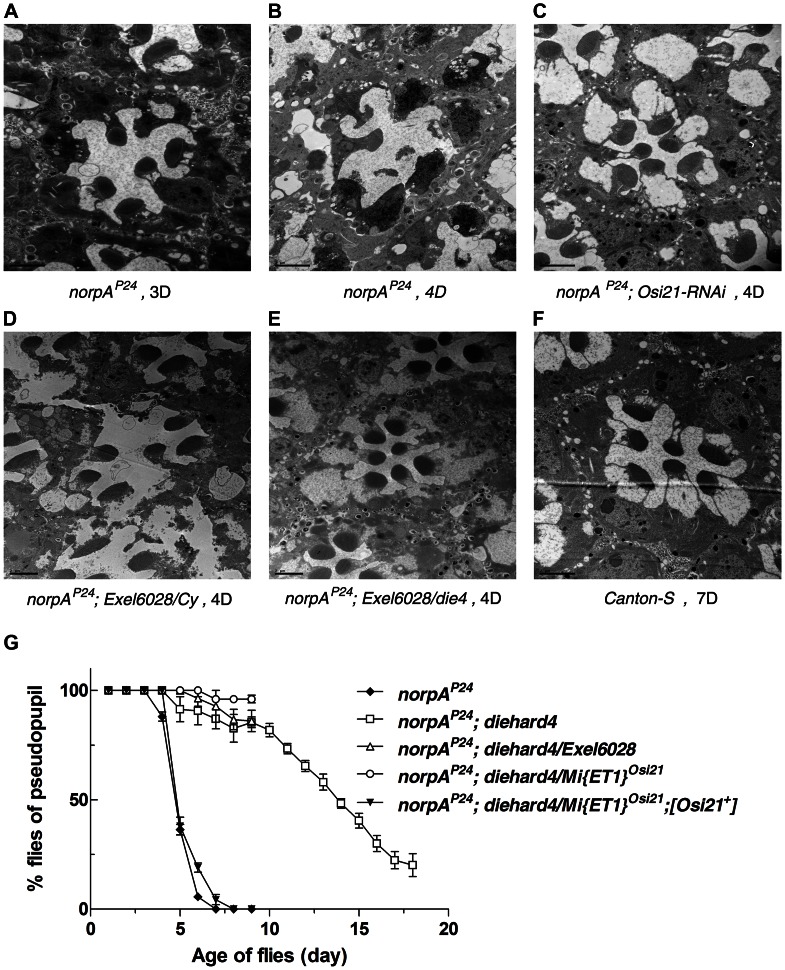
Molecular identification of *die4*. (A–F) Morphology of sectioned ommatidium exposed to constant light was observed by electron microscopy. The *norpA^P24^* flies showed progressive retinal degeneration upon exposure to constant light (A–B). The loss of *Osi21* function by specific knock-down by RNAi rescued retinal degeneration caused by *norpA^P24^* (C). Compared Exel6028 heterozygote (D), retinal degeneration in *norpA^P24^* flies was also rescued by generating transheterozygote flies with genomics deficiency Exel6028 and *die4* (E). Wild-type (Canton-S) flies showed no sign of retinal degeneration upon extended light exposure (F). (G) Newly eclosed flies reared under constant light. The fraction of flies with deep pseudopupil was plotted using approximately 20 flies, in triplicate, of *norpA^P24^*, *norpA^P24^*; *die4*, *norpA^P24^*; *die4/Exel6028*, *norpA^P24^*; *die4/Mi{ET1}^Osi21^*, *norpA^P24^*; *die4/Mi{ET1}^Osi21^*; *[Osi21]^+^*. The *die4/Mi{ET1}^Osi21^* transheterozygote flies were protected from *norpA^P24^* degeneration as *die4/Exel6028* and the protection was reverted by the introduction of the genomic fragment encompassing the *Osi21* gene. Data are shown as the mean ±SE in triplicate. (A) *w, norpA^P24^; Rh1::Gal4/+*, 3 days, (B) *w, norpA^P24^; Rh1::Gal4/+*, 4 days, (C) *w, norpA^P24^; Rh1::Gal4/UAS::Osi21-RNAi* , 4 days, (D) *w, norpA^P24^; Exel6028/Cy*, 4 days, (E) *w, norpA^P24^; die4/Exel6028*, 4 days, (F) Canton-S, 7 days. Light intensity, 2900 lux, Scale bar, 1 µm.

*Drosophila norpA* encodes eye-specific phospholipase C and acts as a central effector in phototransduction [6]. The *norpA* photoreceptor has been used as a model system for studying progressive retinal dystrophies in humans because the loss of its function leads to rapid light-dependent retinal degeneration as a result of excessive endocytosis of stable rhodopsin-arrestin complexes and accumulation of internalized rhodopsin in late endosomes [11], [12], [14]. Previous studies [29] have shown that the *norpA^P24^* (a strong hypomorphic allele of *norpA* [30]) photoreceptor showed progressive retinal degeneration (Figure 1A–B). Its degenerative phenotype appeared within three days and was obvious within four days upon constant light exposure. We found that wild-type (Canton-S) flies showed no sign of retinal degeneration even after seven days of constant light exposure (Figure 1F), indicating that *norpA^P24^* degeneration was strongly dependent on light.

The *die4* mutant was previously identified as a *norpA^P24^* suppressor from a genetic screen by using eye-specific FLP-FRT mosaic flies [31], delaying degeneration several days (Figure 1G). The *die4* mutant was generated using ethyl methanesulfonate (EMS) mutagenesis, possibly bearing multiple mutations. In addition, mosaic screening enables identification of both lethal and non-lethal mutations. Because of these complexities, we used multiple mapping methods to identify the exact mutation responsible for the suppressive phenotype of the *die4* mutant. We previously reported that the mutation in the cytological region of 32D5 to E4 of the *die4* chromosome, is responsible for the suppressive phenotype [31]. Although the *die4* chromosome is homozygous lethal, this mutation is viable, in that the genomic deficiency, Exel6028, failed to complement the suppressive phenotype of *die4*, and was still viable (Table S1).

**Figure 2:**
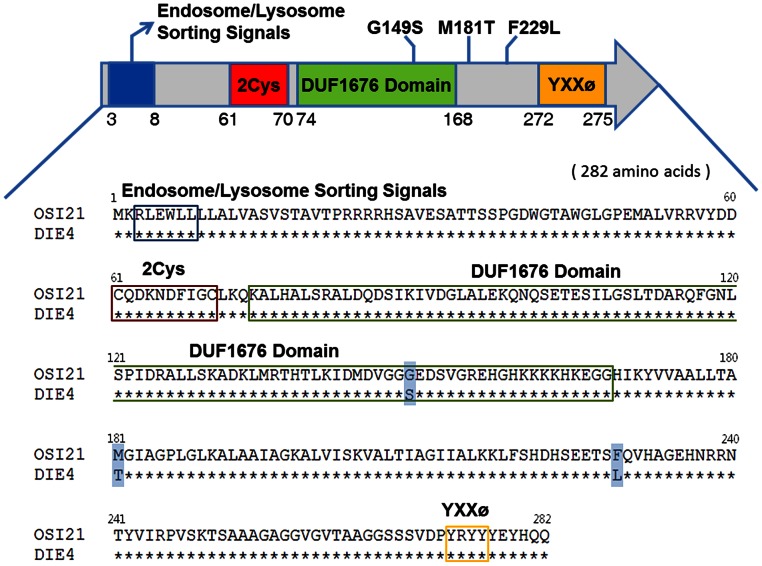
Structural features and sequence of the OSI21 protein. The 282 amino acid long OSI21 protein has at least four distinct motifs/domains, including an endosome/lysosome sorting signal (blue), a 2-Cys region (red), a DUF1676 domain (green), and a YXXØ motif (yellow). Sequencing analysis identified three significant amino acid changes (checked blue) in DIE4, presumably causing a functional loss of *Osi21*.

In this context, we performed a complementation test of *die4* with 11 genes deleted in Exel6028 to identify EMS-induced mutations responsible for the suppressive phenotype (Figure S1A, Table S2). We identified that the loss of *Osi21* (CG14925) is responsible for the suppressive phenotype of *die4*, in that the Minos transposon-inserted allele of *Osi21*, *Mi{ET1}^Osi21^* [32], failed to complement *die4* and the introduction of the genomic fragment encompassing *Osi21* reversed the suppressive effect of *die4/Mi{ET1}^Osi21^* at the deep pseudopupil (DPP) level (Figure 1G, Table S2). Sequence analysis of the *die4* chromosome revealed significant amino acid changes (G149S, M181T, and F229L) in *Osi21* (Figure 2, Figure S1). These results were confirmed by targeted knock-down of *Osi21* using RNAi method (Figure 1C). Therefore, the suppressive effect of *die4* on *norpA^P24^*–triggered retinal degeneration is due to the loss of *Osi21* function. We therefore conclude that *die4* is a loss-of-function allele of *Osi21*.

*Osi21* was identified as an *Osiris* family protein, without known cellular functions, on the basis of sequence homology [26]. Computational analysis, as described by Shah *et al.* [27], was performed using its amino acid sequence, which revealed that OSI21 includes (1) an endosome/lysosome sorting signal, (2) a two-Cys region, (3) duf1676 (Pfam family: PF07898), and (4) a YXXØ motif (Figure 2) as predicted by previous studies [26], [27], [33]. Interestingly, *Osi21* is located on the 2L chromosome. Thus, *Osi21* is not linked to the cluster of 20 Osiris family genes that are located in the Triplo-lethal region (Tpl) of the 3R chromosome and is responsible for the Tpl phenotype, suggesting that its cellular function differs from that of the other *Osiris* family proteins.

### The Loss of *Osi21* Function Suppresses Retinal Degeneration in *norpA^P24^*, *rdgC^306^*, and *trp^1^*, but not in *rdgB^2^* Mutant Photoreceptors

**Figure 3:**
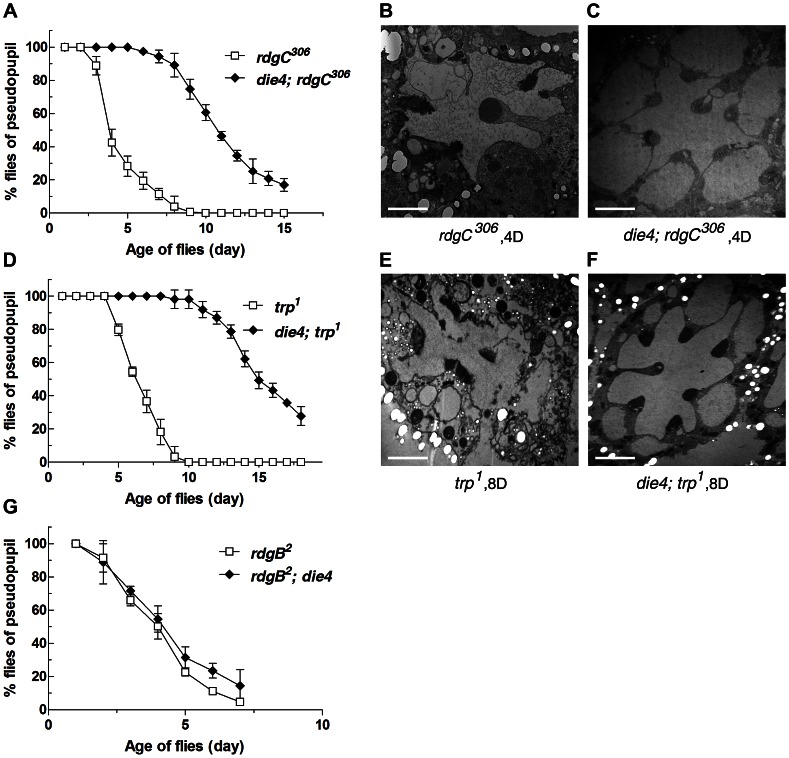
The loss of *Osi21* function suppresses *rdgC^306^*, *trp^1^*, but not *rdgB^2^*. The suppressive effect of *Osi21* loss was examined in deep pseudopupil (DPP) (A, D, G) and electron micrograph level (B–C, E–F). For DPP analysis, newly eclosed flies were exposed to 2900 lux of constant light in the 25°C incubator. Maintenance of DPP was scored each day. The fraction of flies with DPP was plotted using 100 flies of each genotype in triplicate. Data are shown as the mean ±SE. (A) *rdgC^306^* and *die4; rdgC^306^*, (D) *trp^1^* and *die4; trp^1^*, and (G) *rdgB^2^* and *rdgB^2^; die4*. For electron micrograph, flies in each genotype were exposed to 2900 lux of constant light in the 25°C incubator. Four-day old *rdgC^306^* (B) and *die4; rdgC^306^* (C), Eight-day old *trp^1^* (E) and *die4; trp^1^* (F) were sacrificed for analysis. Compared to the completely deformed ommatidial structure of single mutants, double mutants showed well preserved rhabdomeric and ommatidial structures.

*Drosophila rdgC* encodes rhodopsin-specific phosphatase and requires rhodopsin recycling [34], [35]. The loss of *rdgC* function leads to light- and age-dependent retinal degeneration as a result of excessive endocytosis of stable rhodopsin-arrestin complexes [14]. Because the *rdgC* mutant photoreceptor cells share the cause of retinal cell death with *norpA^P24^*, we used *rdgC^306^*, the loss-of-function *rdgC* mutant, to test the effect of *die4* on retinal cell death. DPP analysis and histological analysis using electron microscopy showed that *die4* protects retinal degeneration due to *rdgC^306^* (Figure 3A–C, Figure S2), suggesting that *Osi21* is not a specific regulator in *norpA*-triggered retinal degeneration but plays an essential role in retinal degeneration caused by intracellular accumulation of cytotoxic rhodopsin.

We also examined the effect of *die4* on retinal degeneration due to the loss of *trp* function, a light-sensitive Ca^2+^ channel [36]. *Drosophila trp^1^* was recovered as a spontaneously occurring temperature-sensitive loss-of-function mutant at a temperature of 24°C [36], [37] and is known to show light-enhanced retinal degeneration [38]. Although a dysfunction in Ca^2+^ fluctuation was suggested as a cause of its retinal degeneration phenotype, its mechanism of degeneration remains unknown. Interestingly, we found that the loss of *die4* function protects retinal degeneration caused by *trp^1^* at the DPP and ultrastructural levels (Figure 3D–F, Figure S2). This result suggests that intracellular accumulation of cytotoxic rhodopsin also causes retinal degeneration in *trp^1^* mutant photoreceptor cells.

We used *rdgB^2^* photoreceptors as a negative control to assume the functions of *die4* because cytoplasmic rhodopsin aggregation is not involved in retinal degeneration in *rdgB^2^* photoreceptors [39]. As expected, *die4* was unable to suppress *rdgB^2^*-triggered retinal degeneration (Figure 3G). Combined together, these double mutant analyses suggest that intracellular rhodopsin aggregation triggers unregulated cell death in *norpA^P24^*, *rdgC^306^*, and *trp^1^* photoreceptors, and that *Osi21* is a key regulator in the formation of rhodopsin aggregation, wherein the loss of *Osi21* function suppresses retinal degeneration in these mutant photoreceptor cells.

### *Osi21* Negatively Regulates Late Endosomal Membrane Traffic toward Lysosomes

Previous studies found that *norpA* and *rdgC* mutant photoreceptor cells die due to excessive endocytosis of rhodopsin-arrestin complexes and accumulation of endocytosed rhodopsin in late endosomes [11], [12], [14]. These findings indicate that the inability of rhodopsin transport and degradation through the endo-lysosomal system cause unregulated cell death in *norpA* and *rdgC* mutant photoreceptors. In this context, we tested the possibility that *Osi21* acts as a regulator that maintains membrane homeostasis between endosomes and lysosomes in which the functional loss of *Osi21* shifts the membrane balance of the endo-lysosomal system.

**Figure 4:**
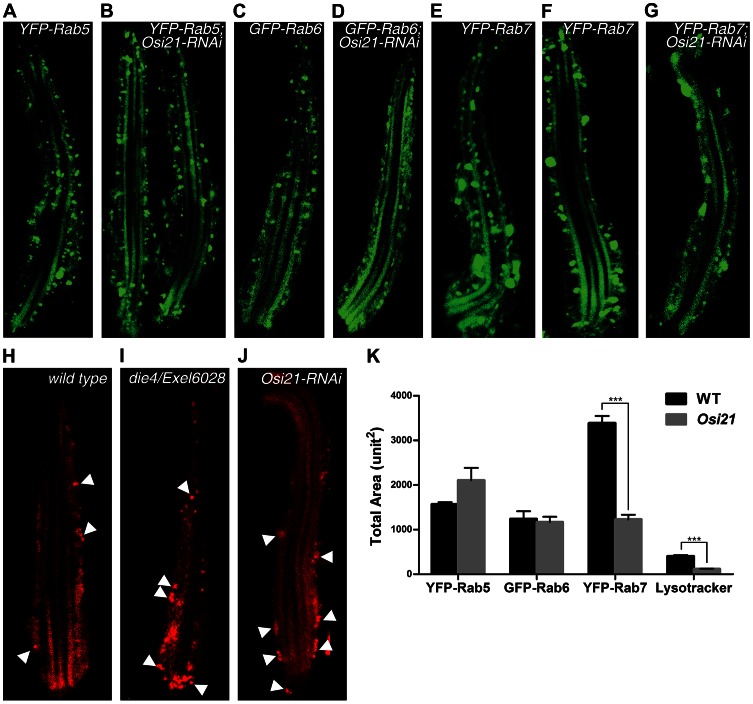
The loss of *Osi21* function modulates the membrane balance of the endo-lysosomal system. A single ommatidium was prepared from newly eclosed fly retina and was visualized by confocal microscopy. Each subcellular structure was marked with YFP-Rab5 (early endosomes, A–B), GFP-Rab6 (Golgi complexes, C–D), YFP-Rab7 (late endosomes, E–G), and Lysotracker (lysosomes, H–J). (A–B) Compared to the wild-type photoreceptor cell (A), the *Osi21* knock-down photoreceptor (B) showed slightly reduced levels of Rab5-positive vesicles (early endosomes) both in size and number. (C–D) No significant differences in Rab6-positive vesicles (Golgi complexes) were observed between the wild-type photoreceptor (C) and the *Osi21* knock-down photoreceptor (D) both in size and number. (E–G) Compared to the wild-type photoreceptor cell (E, F), the *Osi21* knock-down photoreceptor (G) showed greatly reduced levels of Rab7-positive vesicles (late endosomes) both in size and number. (H–J) Compared to the wild-type photoreceptor cell (H), the *Osi21* loss-of-function photoreceptor showed proliferated Lysotracker staining (lysosomes) (I–J). Significant difference only in lysosomal number, but not size, between the wild-type (H) and the *Osi21* loss-of-function photoreceptor (I–J) was observed. (K) Total area marked with the vesicles was quantified from three representative photoreceptor cells of each genotype in triplicate. Error bars indicate SEM. ***p<0.01. (A) *w; Rhi1::Gal4, UAS::YFP-Rab5/+*, (B) *w; Rhi1::Gal4, UAS::YFP-Rab5/+; UAS::Osi21-RNAi/+*, (C) *w; Rhi1::Gal4, UAS::YFP-Rab6/+*, (D) *w; Rhi1::Gal4, UAS::YFP-Rab6/+; UAS::Osi21-RNAi/+*, (E) *w; Rhi1::Gal4, UAS::YFP-Rab7/+*, (F) *w; Rhi1::Gal4, UAS::YFP-Rab7/+; UAS::RFP-arf72A/+*, (G) *w; Rhi1::Gal4, UAS::YFP-Rab7/+; UAS::Osi21-RNAi/+*, (C) *w*, (D) *die4/Exel6028*, (E) *Rh1:Gal4/UAS::Osi21-RNAi/+*. (arrows) Rab7-positive late endosomes (arrowheads) lysosomes. Flies were kept in the fly culture room, maintaining 25°C, 18 h light/8 h dark cycles.

To test our hypothesis, we examined whole mounts of *Drosophila* retinas by confocal microscopy. We found that the loss of *Osi21* function minimally affected the Rab5-positive vesicles (early endosomes) (Figure 4A–B) and didn't affect the Rab6-positive vesicles (Golgi complexes) (Figure 4C–D). However, the loss of *Osi21* function significantly affected the Rab7-positive vesicles (late endosomes). Compared to the wild-type photoreceptor cells (Figure 4E–F), both size and number of Rab7-positive vesicles were greatly reduced in *Osi21* knock-down photoreceptor cells (Figure 4G). Accordingly, lysosomal compartments proliferated in *Osi21* knock-down photoreceptor cells (Figure 4I–J), suggesting that the membrane balance of endo-lysosomal trafficking shifted toward lysosomes. Interestingly, the loss of *Osi21* function only affected the number, but not size, of the lysosomal compartments in *Osi21* knock-down photoreceptors, thus reflecting limited lysosomal rhodopsin flow in newly eclosed flies.

The reduced Rab7-positive vesicles in *Osi21* knock-down photoreceptor cells may be attributed to Gal4 titration due to the existence of a second UAS promoter of in the *Osi21* knock-down construct. Thus, we used the *w; Rhi1::Gal4, UAS::YFP-Rab7/+; UAS::RFP-arf72A*/+ as a titration control and showed that the second UAS promoter did not affect YFP-Rab7 expression (Figure 4F). Quantification of each vesicle clearly showed that among the Rab5-, the Rab6- and the Rab7-positive vesicles, the loss of *Osi21* function only affected the Rab7-positive vesicles (Figure 4K). Minimal increase of the Rab5-positive area in *Osi21* knock-down photoreceptors may be the secondary effect caused by a reduction in Rab7-positive vesicles. Accordingly, biochemical analysis of isolated endo-lysosomal vesicles using Iodixanol density gradients (See Text S1) showed that immunoreactivities of Rab7, which was colocalized with endocytosed rhodopsin, were shifted toward the lower density fractions by the loss of *Osi21* function (Figure S3), suggesting a reduced fraction of late endosomes in Rab7-positive vesicles [40], [41]. These results suggest that the loss of *Osi21* function specifically affects the membrane balance between late endosomes and lysosomes.

**Figure 5:**
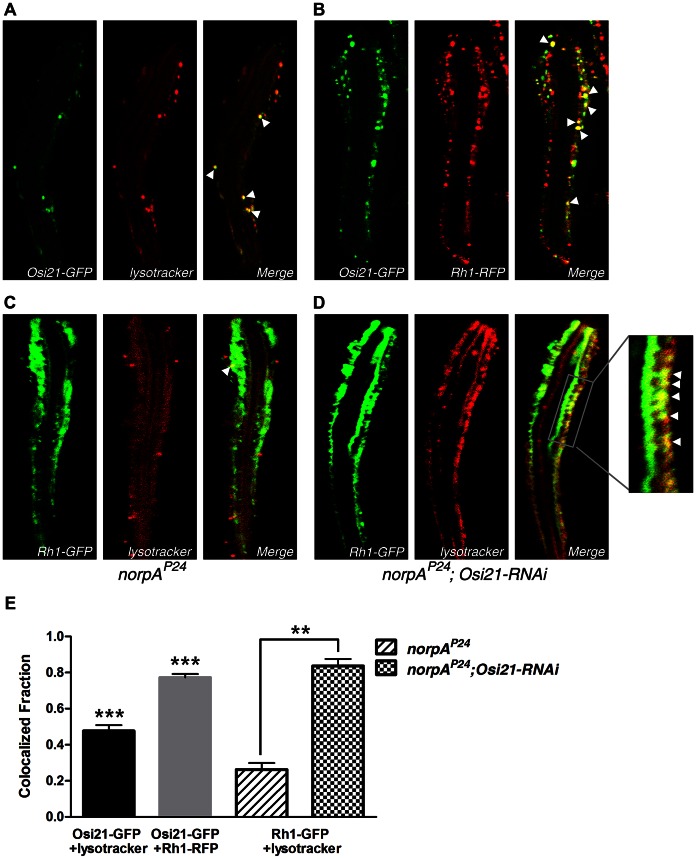
OSI21 partially localizes on the endo-lysosomal system. Newly eclosed flies were exposed to bright light (2900 lux) for 90 min. A single ommatidium prepared from fly retina was visualized by confocal microscopy. (A–B) The OSI21 protein was marked with GFP. Each subcellular structure was marked with Lysotracker -Red (lysosomes, A) and Rhodopsin-RFP (endosomes, B). OSI21 partially localizes on lysosomes (Pearson's correlation coefficient: 0.617, A). OSI21 partially localizes on the rhodopsin positive vesicles (Pearson's correlation coefficient: 0.635, B). (A) *w; Rhi1::Gal4, UAS::Osi21-GFP/+* (B) *w; Rhi1::Gal4, UAS::Osi21-GFP/+; UAS::Rh1-RFP/+*. (arrowheads) lysosomes or endosomes colocalized with OSI21-GFP. Flies were kept in the 25°C fly culture room, 18 h light/8 h dark cycles. (C–D) Localization of endocytosed rhodopsin with lysosomes. Newly eclosed flies were exposed to bright light (2900 lux) for 90 min. A single ommatidium was prepared from fly retina and examined using confocal microscopy. GFP-labeled rhodopsin and Lysotracker -red were used for visualizing rhodopsin endocytosis and lysosomes. Lysosomes (red) in *norpA^p24^* photoreceptor cells are smaller in number and are not overlapped with endocytosed rhodopsin (green) (Pearson's correlation coefficient: 0.342, C). (Arrowhead in C) Endocytosed rhodopsin escaped *Osi21* blockage, reflecting regular lysosomal rhodopsin-turnover. On the other hand, lysosomes are greatly proliferated and are colocalized with endocytosed rhodopsin in the *norpA^p24^* mutant photoreceptor with a *Osi21-RNAi* transgene (Pearson's correlation coefficient: 0.604, D). (Arrowheads) Lysosomes collocalized with endocytosed rhodopsin. (C) *norpA^p24^; Rh1::Gal4, UAS::Rh1-GFP/+* (D) *norpA^p24^; Rh1::Gal4, UAS::Rh1-GFP/UAS::Osi21-RNAi*. (E) Relative number of green-labeled vesicles among red-labeled population was calculated from three representative photoreceptor cells of each genotype in triplicate. Error bars indicate SEM. ***p<0.01, **p<0.05.

Because the specific shift of membrane balance between late endosomes and lysosomes raised a strong possibility of direct regulation of *Osi21* on membrane homeostasis of the endo-lysosomal system, we examined the subcellular localization of the OSI21 protein. Newly eclosed flies were reared in a light/dark cycled incubator and were exposed to bright light (2900 lux) for 90 min to induce massive rhodopsin endocytosis and its accumulation in late endosomes. The subcellular localization of OSI21-GFP from whole mount ommatidia was examined using confocal microscopy. We assumed that the OSI21-GFP is functional because its expression counterbalanced the suppressive effect of *Osi21-RNAi* in the DPP level (Data not shown). We found the OSI21-GFP localization partially overlapped with Lysotracker staining (Figure 5A and E, Pearson's correlation coefficient: 0.617). In addition, majority of Osi21-GFP colocalized with endocytosed Rh1-RFP (Figure 5B and E, Pearson's correlation coefficient: 0.635), suggesting that *Osi21* functions directly on the endo-lysosomal membrane system in a way that *Osi21* negatively regulates late endosomal membrane traffic toward lysosomes, resulting in rhodopsin accumulation in late endosomal compartments.

### The Loss of *Osi21* Function Results in the Facilitated Degradation of Endocytosed Rhodopsin

Changes in membrane balance between late endosomes and lysosomes may also affect the dynamics of vesicular traffic and the rate of rhodopsin degradation, in which the loss of *Osi21* function facilitates rhodopsin traffic toward lysosomes and its lysosomal degradation, resulting in a delay of retinal degeneration in *norpA^P24^* photoreceptor cells. In fact, reduced rhodopsin content due to vitamin A deprivation or mutation in the rhodopsin gene rescued *norpA*-triggered retinal degeneration [42]. Our analysis by confocal microscopy showed that, compared to the *norpA^P24^* photoreceptor cells (Figure 5C), *norpA^P24^* mutant photoreceptor cells with the *Osi21-RNAi* transgene showed greatly proliferated lysosomes (Figure 5D–E). These lysosomes were colocalized with endocytosed rhodopsin (Pearson's correlation coefficient: 0.604). Although we often found that small amounts of endocytosed rhodopsin escaped Osi21 blockage (Figure 5C, arrowhead) and was colocalized with the lysosome (Pearson's correlation coefficient: 0.342), there were less lysosomes in the control *norpA^P24^* photoreceptor cells. Moreover, majority of lysosomes did not colocalized with endocytosed rhodopsin, indicating that such colocalization reflected regular lysosomal turnover. These results raise the possibility that the loss of *Osi21* function facilitates the rhodopsin degradation in lysosomes.

**Figure 6:**
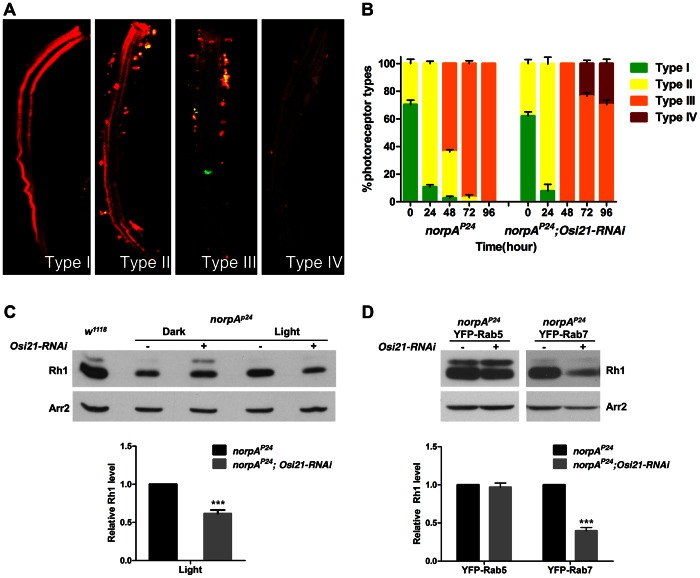
The loss of *Osi21* function facilitates rhodopsin degradation by modulating the endo-lysosomal membrane dynamics in light-induced *norpA^p24^* photoreceptors. (A–B) Rate of rhodopsin endocytosis and degradation. Movement of light-pulsed RFP-tagged rhodopsin from the rhabdomere was examined by confocal microscopy. Observed photoreceptors were categorized according to the Rh1 localization. (A) Photoreceptor types. Type I (Rh1-RFP is localized in the rhabdomere), Type II (Rh1-RFP is localized in the rhabdomere as well as cytoplasm), Type III (Rh1-RFP is localized in the cytoplasm) and Type IV (Rh1-RFP disappears due to degradation). (B) Percentage of each typed photoreceptor cells was calculated at 24 h intervals for 96 h. In each time point, approximately 30 photoreceptor cells of each genotype from five individuals were examined. The initial movement of endocytosed rhodopsin toward the endosomal system (Type I and the Type II) was not different between the *norpA^P24^* and *norpA^P24^*; *Osi21-RNAi* photoreceptor. However, facilitated clearance of endocytosed rhodopsin (Type IV) in *norpA^P24^* mutant photoreceptor cells with the *Osi21-RNAi* transgene was observed. Error bars indicate the SEM. p<0.01 (the Kolmogorov-Smirnov test). (C) The loss of *Osi21* function in *norpA^p24^* flies leads to the reduced rhodopsin. Flies were reared either in the dark to prevent rhodopsin endocytosis or in the 18 h light/8 h dark cycles to stimulate rhodopsin endocytosis, and were collected within 12 h after eclosion. Western blot analyses were performed to measure the effect of the *Osi21* functional loss on the degradation of endocytosed rhodopsin. Relative Rh1 level was calculated from triplicated immunoblots. Data are shown as the mean ±SE. ***p<0.01. *w^1118^* (lane1), *norpA^p24^; Rh1::Gal4/+* (dark-reared, lane2) , *norpA^p24^; Rh1::Gal4/+; UAS:: Osi21-RNAi/+* (dark-reared, lane3) , *norpA^p24^; Rh1::Gal4/+* (18 h light/8 h dark, lane4) , *norpA^p24^; Rh1::Gal4/+; UAS:: Osi21-RNAi/+* (18 h light/8 h dark, lane5). (D) Overexpression of Rab7 synergistically affects rhodopsin degradation with the loss of *Osi21* function. Newly eclosed flies were exposed to bright light (2900 lux) for 48 h before being sacrificed to induce the maximal rhodopsin endocytosis. Western blot analyses were performed to measure the effect of the *Osi21* functional loss on the degradation of endocytosed rhodopsin in response to the activation of the endosomal trafficking machinery. Relative Rh1 level was calculated from triplicated immunoblots. Data are shown as the mean ±SE. ***p<0.01. *norpA^p24^; Rh1::Gal4, UAS:: YFP-Rab5/+* (lane1) , *norpA^p24^; Rh1::Gal4, UAS:: YFP-Rab5/+; UAS:: Osi21-RNAi/+* (lane2) , *norpA^p24^; Rh1::Gal4, UAS:: YFP-Rab7/+* (lane3) , *norpA^p24^; Rh1::Gal4, UAS:: YFP-Rab7/+; UAS:: Osi21-RNAi/+* (lane4).

In this context, first, we examined the rate of rhodopsin endocytosis and degradation by time course measurements of pulse-chased photoreceptors by confocal microscopy. For the measurements, we expressed RFP-tagged rhodopsin under the control of the hs::Gal4 driver. Newly eclosed *norpA^P24^* and *norpA^P24^*; *Osi21-RNAi* flies were kept in complete darkness for 24 h and then subjected to heat-shock. These flies were then kept in the darkness for another day to allow synthesis and transport of Rh1-RFP to the rhabdomere, following which they were exposed to bright light to chase Rh1-RFP. Whole mount photoreceptors were examined by confocal microscopy at 24 h interval for 96 h by typing the photoreceptor based on the progression of rhodopsin endocytosis and degradation (Figure 6A). We found that the initial movement of endocytosed rhodopsin toward the endosomal system was not different between the *norpA^P24^* and *norpA^P24^*; *Osi21-RNAi* photoreceptor (Figure 6B, see the percentage of Type I and Type II photoreceptors). However, *norpA^P24^* mutant photoreceptor cells with the *Osi21-RNAi* transgene showed facilitated clearance of endocytosed rhodopsin (Figure 6B, see the percentage of Type III and Type IV photoreceptors), indicating facilitated degradation of endocytosed rhodopsin through the loss of *Osi21* function.

The effect of the *Osi21* loss-of-function on the rhodopsin contents in *norpA^P24^* photoreceptors was also examined by western blot analysis by using flies eclosed within 12 h. Flies were reared either in the dark to prevent rhodopsin endocytosis or in 18 h light/8 h dark cycles to stimulate rhodopsin endocytosis. No significant differences in rhodopsin content were observed in the dark-reared *norpA^P24^* mutant photoreceptor cell with the *Osi21-RNAi* transgene, compared to dark-reared *norpA^P24^* photoreceptors (Figure 6C, lanes 2–3). However, the rhodopsin content was greatly reduced in the *norpA^P24^* mutant photoreceptor cells with the *Osi21-RNAi* transgene by the bright light stimulation (Figure 6C, lanes 4–5). These results suggest that more endocytosed rhodopsin was transported into and degraded by lysosomes because of *Osi21* loss-of-function.

Massive influx of Rh1 into the endosomal system may saturate endosomal trafficking machinery, resulting in the late endosomal accumulation of endocytosed rhodopsin. Therefore, we tested the effect of the *Osi21* loss-of-function on rhodopsin content when the endosomal trafficking machinery was activated by overexpressing Rab5 or Rab7 (Figure S4). To induce the maximal rhodopsin endocytosis, newly eclosed flies were exposed to bright light (2900 lux) for 48 h. No significant influence of Rab5 overexpression on the degradation of endocytosed rhodopsin was observed (Figure 6D, left). However, overexpression of Rab7 synergistically accelerated rhodopsin degradation with the loss of function of *Osi21* (Figure 6D, right). Considering no significant decrease in rhodopsin content was observed with Rab7 overexpression alone, our results suggest that *Osi21* negatively regulated rhodopsin transport between late endosomes and lysosomes by interacting with the Rab7-positive trafficking machinery. Therefore, we conclude that *Osi21* is a critical negative regulator of vesicular traffic between endosomes and lysosomes. Its functional loss suppresses retinal degeneration in phototransduction mutants by changing the membrane dynamics between late endosomes and lysosomes and by facilitating the degradation of endocytosed rhodopsin.

## Discussion

In both vertebrates and invertebrates, malfunctioning of phototransduction may stimulate the cell death machinery, resulting in retinal degeneration [43]. Without functional phototransduction, rhodopsin-1, the major visual pigment, is rapidly endocytosed and accumulated in the late endosomes [12]. Impaired lysosomal delivery of endocytosed rhodopsin and its degradation trigger progressive and light-dependent retinal degeneration in phototransduction mutants [12], [17]. However, the mechanism underlying the accumulation of endocytosed rhodopsin in late endosomes, instead of delivering to lysosomes for degradation, remains to be elucidated.

**Figure 7:**
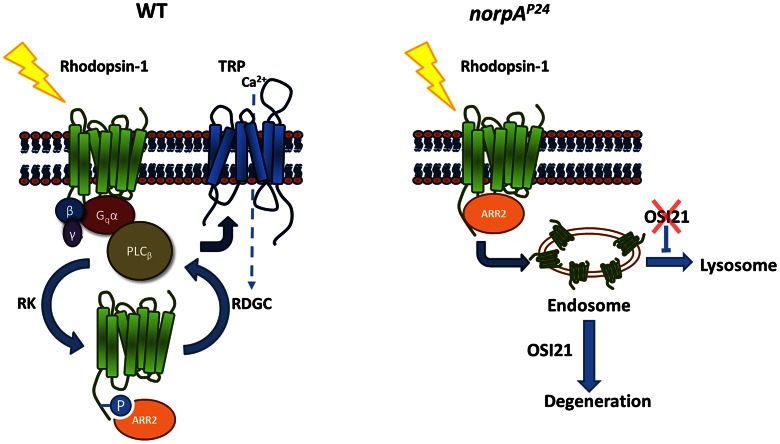
A model for Osi21 function on photoreceptor degeneration in phototransduction mutants. (Left) In the wild-type photoreceptor, light-activated rhodopsin initiates phototransduction, relays the signal through PLC_β_ (*norpA*), and induces Ca^2+^ influx through the cation-specific calcium channel (*trp*). On the other hand, rhodopsin kinase (RK) rapidly phosphorylates the activated rhodopsin, enabling arrestin (Arr2) binding for inactivation. The Rh1-Arr2 complex requires a specific calcium-dependent phosphatase (rdgC) activity for dissociation. Stable rhodopsin-arrestin complex due to the lack of Ca^2+^ influx results in massive endocytosis, presumably underlying retinal degeneration in the *norpA^p24^*, *rdgC^306^*, and *trp^1^* mutant photoreceptor. (Right) In *norpA^p24^* photoreceptors, the formation of stable rhodopsin-arrestin complexes results in its massive endocytosis and accumulation of endocytosed rhodopsin in late endosomes. *Osi21*may result in the inability of rhodopsin transport to the lysosome for degradation by negatively regulating endo-lysosomal flow.

In the current study, we used *die4*, the *norpA^P24^* suppressor, to investigate the molecular basis of the accumulation of rhodopsin in late endosomes in phototransduction mutants. We found that the loss of *die4* function delays retinal degeneration in *norpA^P24^*, *rdgC^306^* and *trp^1^*, but not in *rdgB^2^*. These results suggest that, at least, *norpA^P24^*, *rdgC^306^*, and *trp^1^* photoreceptor cells die through a shared route. Previous research suggested that the generation of stable rhodopsin-arrestin complexes is the major cause of cell death in *norpA^EE5^* [11] and *rdgC^306^* [14]. The formation of stable rhodopsin-arrestin complexes in the *norpA* mutant photoreceptor is attributable to its inability to activate the calcium-dependent phosphatase, RDGC, which dephosphorylates rhodopsin (Figure 7). The calcium-dependent phosphatase also remains inactive in the *trp^1^* photoreceptor upon light exposure since the cation specific calcium channel is lost in *trp^1^* [36]. Therefore, all three phototransduction mutants share a common feature; the formation of stable rhodopsin-arrestin complexes.

On the other hand, *norpA^P24^*, *rdgC^306^* and *trp^1^* require light activation of rhodopsin but not subsequent phototransduction for retinal degeneration [44]. In contrast, *rdgB^2^* requires both, whereby (1) *rdgB^2^* flies fail to degenerate in complete darkness [44], (2) the *rdgB^2^* retinal degeneration is rescued by *norpA^P24^* [44], and (3) the *rdgB^KS222^* retinal degeneration is rescued by *trp^1^* [38]. These findings are used to infer that *rdgB^2^* photoreceptor cells die via a different route.

We found that the loss of *die4* function delays retinal degeneration in *norpA^P24^* longer than those in *rdgC^306^* and *trp^1^*. These results suggest that the blockage of endo-lysosomal trafficking by *Osi21* is not the sole cause of retinal degeneration in *rdgC^306^* and *trp^1^* mutants. Recently, Sengupta *et al.* [45] proposed that PI(4,5)P_2_ depletion by NORPA underlies retinal degeneration in *trp^CM^* and *trp^343^* mutants. Interestingly, both mutants exhibit faster light-dependent retinal degeneration than *trp^1^* mutants. Preventing the formation of stable Rh1-Arr2 complexes by red light slows down the retinal degeneration in *trp^CM^* and *trp^343^* mutants comparable to the *trp^1^* degeneration in white light, suggesting that the endocytosis of Rh1-Arr2 complexes contributes retinal degeneration in *trp* mutants and different results are attributable in part to the allelic differences. In addition, PI(4,5)P_2_ depletion affects arrestin-mediated endocytosis [45], so that Rh1 internalization might be reduced in *trp* mutants. However, their results raise a strong possibility that prolonged activation of NORPA possibly contributes to degenerative syndromes in both *trp* and *rdgC* mutants.

Double mutant photoreceptor cells are eventually degenerated; they lost their DPP with extended exposure to bright light. Although DPP analysis does not provide a measure of the retinal degeneration process, it faithfully measures a complete loss of the ommatidial structure as it reaches the end of the degenerative process. DPP analysis in the current study suggests that the loss of *Osi21* function delays the onset of retinal degeneration in *norpA^P24^*, *rdgC^306^* and *trp^1^* mutants. However, the loss of *Osi21* function slows down the retinal degeneration in *norpA^P24^*, but not in *rdgC^306^* and *trp^1^*: the slope of DPP loss was similar to that of the control soon after DPP loss occurred in double mutants. These results also suggest that the activity of *Osi21* is not the sole cause of the *rdgC* and the *trp* degeneration.

We conclude that *Osi21* acts as a negative regulator of endo-lysosomal membrane traffic between late endosomes and lysosomes. This conclusion is based on the following observations: (1) Both the size and number of the late endosomes are significantly reduced in *Osi21* knock-down photoreceptor cells, (2) the lysosomal compartments are greatly proliferated in *Osi21* knock-down photoreceptor cells, (3) the OSI21 protein is localized in the endo-lysosomal compartments, (4) the loss of *Osi21* function in the *norpA^P24^* mutant photoreceptor facilitates the degradation of endocytosed rhodopsin, and (5) Rab7 overexpression alone fails to affect the rhodopsin content of the *norpA^P24^* photoreceptor. However, overexpression of Rab7 synergistically accelerates rhodopsin degradation with the loss of *Osi21* function, suggesting that *Osi21* directly interacts with the Rab7-positive trafficking machinery. These results clearly demonstrate that the existence of negative blockage regulating the membrane balance of the endo-lysosomal system, and not the capacity of endo-lysosomal trafficking machinery, causes retinal degeneration in phototransduction mutants.

Heptahelical G protein-coupled receptors (GPCRs) are considered the most diverse and therapeutically important family of receptors [46], [47]. Like many vertebrate GPCRs, light-activated rhodopsin-1 in *Drosophila* is rapidly phosphorylated by a specific kinase, called rhodopsin kinase. Phosphorylated rhodopsin-1 is desensitized by arrestins and is endocytosed to terminate further signaling activity (Figure 7). Because of this similarity, *Drosophila* rhodopsin-1 has been used as a prototype to study agonist-induced desensitization and internalization of vertebrate GPCRs [48].

In *Drosophila* photoreceptors, Arr2 promotes rhodopsin endocytosis and degradation when stable Rh1-Arr2 complexes are generated by loss of *norpA* or *rdgC* while Arr1 promotes rhodopsin endocytosis and recycling in the normal condition [14], [49]. Although Arr1 was previously reported to localize in endosomes [49], we found that Arr2 was absent in the endosomal system (data not shown), indicating Arr2 dissociates from Rh1 near the rhabdomeric membrane. This is reminiscent of functional classification of vertebrate GPCRs: Class A and Class B [50]–[52]. Thus, it can be clearly surmised that *Drosophila* photoreceptors operate two separate mechanisms of Rh1 endocytosis: (1) Arr1 for quenching and recycling, and (2) Arr2 for quenching and degradation. Since Arr2 is several folds more abundant than Arr1 in *Drosophila* photoreceptor cells to ensure rapid quenching of rhodopsin signaling for visual sensitivity [53], [54], the negative blockage by *Osi21* may be evolved to counterbalance excessive Rh1 degradation as a result of Arr2 binding to activated Rh1. Therefore, it should be further investigated whether arrestins play roles in the decision between recycling and degradation for endosomal Rh1, and in the activation of cell death machinery.

Post-endocytic trafficking of GPCRs implicates in many human diseases [55]. Especially, stable rhodopsin-arrestin complexes in vertebrates also result in photoreceptor degeneration [56], [57]. In addition, cytoplasmic accumulation of proteins often implicates various neurodegenerative disorders, including the accumulation of rhodopsin in retinitis pigmentosa [56] and the accumulation of polyQ-expanded huntingtin in Huntington's disease [58]. Our results suggest that *Osi21* regulation may underlie accumulation of disease-causing proteins in the endosomal compartment and that the elimination of *Osi21* regulation may clean up this pathologic accumulation. Therefore, the identification and characterization of this specific cellular machinery may provide a therapeutic target for many GPCR-related human diseases and neurodegenerative disorders.

## Materials and Methods

### *Drosophila* Strains and Genetics

*Drosophila* was grown on standard food in a 25°C incubator. Standard genetic schemes were used to generate flies with the genotypes described. The Canton-S and *w^1118^* fly were used as a wild-type strain, *norpA^P24^*, *rdgC^306^*, *trp^1^* and *rdgB^2^* as loss-of-function strains of phototransduction. Genomic deficiencies listed in Table S1. Loss-of-function mutants used for complementation test listed in Table S2. A *w norpA^p24^ eyFLP* chromosome was made using meiotic recombination to subsequently generate *die4* mosaic flies in the *norpA^p24^* background. *P[ry+; w+]30C, P[ry+; hs-neo; FRT]40A*, and *P[w +]70C FLP* recombinase target (*FRT*), *die4* chromosomes and *FRT40A* GMR-*hid* were used in combination with the *w norpA^p24^ eyFLP* chromosome to make photoreceptor cells exclusively homozygous for the *die4 FRT* chromosome [59]. The second and third chromosome inserts of the *Rh1*::*GAL4* driver were derived from the *Rh1::GAL4* line constructed by Tabuchi et al. [60] and used for driving expression of various UAS targets including the *die4* knock-down construct, *UAS::Osi21-RNAi* and fluorescently subcellular markers, *UAS::YFP-Rab5*, *UAS::YFP-Rab7* and *UAS::GFP-Rab6*. *UAS::Rh1-GFP* was constructed using the *prh1::eGFP* construct from Pichaud and Desplan [61]. All *Drosophila* stocks except *Osi21* knockdown strain were obtained from the Bloomington Stock Center at Indiana University. The *UAS::Osi21-RNAi* strain was obtained from Vienna *Drosophila* RNAi Center (VDRC, Vienna).

### Generation of Transgenic Animals

Primers for *Osi21* or *ninaE* (*Rh1*) were specifically designed for use in the Gateway system (Invitrogen, Inc., Carlsbad, CA). Exact primer sequences for the rescue experiment, the expression of GFP-tagged *Osi21* or RFP-tagged *Rh1* were listed in Table S3. Directional cloning into the pENTR TOPO vector and the destination vectors (Carnegie Institution of Washington) followed manufacturer's instruction. The pTW, pTWG, and pTWRvector were used as destination vectors for Rescue constructs, *UAS::Osi21-GFP*, and *UAS::Rh1-RFP*, respectively. Plasmid isolation was performed from positive clones using the Qiagen Midi Kit (Valencia, CA). After injection, G0 flies were crossed with *w*; *SM1/Sco; TM2/Sb* balancer flies. The progeny from the cross were sorted for mini-*w^+^* eyes. Mini-*w^+^* was used as a marker to determine the presence of the transgene. Flies with the mini-*w^+^* eye (G1 generation) were subsequently crossed with *w^1118^* flies to map the location of the transgene. After mating with *w^1118^* flies, mini-*w^+^* flies were crossed to the driver stock. In all cases, mini-*w^+^* flies were crossed to *w; Rh1::GAL4* to drive expression of the transgene.

### Deep Pseudopupil Analysis

The deep pseudopupil (DPP) was visualized in red-eyed flies which shows a bright trapezoidal structure when a white light illuminates the retina from the back of the head [62]. The flies of each genotype were collected daily and raised under the appropriate light condition. The flies were also scored daily for the presence of the deep pseudopupil. During degeneration, deep pseudopupils become increasingly diffused before being completely lost. The deep pseudopupil was scored as negative as soon as its trapezoidal shape became indistinct. The percentage of flies that retained their deep pseudopupils for a given day was calculated. In figure 1, total 20 flies were analyzed for each genotype tested. In figure 2, three replicates with a total of 100 flies were analyzed for *rdgC^306^*, *trp^1^* and *rdgB^2^* with or without *die4* to determine the average percentage of deep pseudopupil -positive flies and the standard error for each day.

### Whole-mount Preparation of *Drosophila* Ommatidia and Fluorescence Microscopy

Detailed procedures were also described previously [63]. Flies eclosed within 6 h were sacrificed with or without light treatment. For the whole-mount ommatidia isolation, fly heads were removed from bodies. A sagittal cut was made on the fly head creating two halves. The brain and proboscis were then removed. The eye was placed on a microscope slide containing 1× PBS. For Lysotracker (Invitrogen, Inc., Carlsbad, CA) staining, fly eyes were preincubated in ∼1 µM Lysotracker for 90 min and washed 3 times in 1× PBS for 30 min. Then, 2% paraformaldehyde in 1× PBS was used as a fixative and treated for 30 min followed by twice wash in 1× PBS. Residual pigments in fly retina were eliminated in 0.1% Triton X-100 (Sigma, MO) for 4 h, then washed twice for 10 min. Each ommatidia were removed using a sharp platinum needle from fly retina. Usually, a large piece of retina was then teased apart and mounted using mounting medium (Vector Labratories, Inc., Burlingame, CA). The FV500 confocal laser scanning microscope (Olympus Optical, Japan) was used for examining individual ommatidium. Optical images were acquired with an ×100 objective. Confocal images were analyzed with ImageJ software (NIH, MD) for quantitative analysis. For quantitative measurement of endosome/lysosome, mean size, number and total area of each vesicle in a photoreceptor cell were calculated with the Analyze Particle function. Measured values were normalized with the known distance option of imageJ. Statistical significances were calculated with two-tailed t tests using Prism 5.01 software. For colocalization analysis, Pearson's correlation coefficient (Rr) was calculated with Intensity colocalization analysis function of imageJ. The values for Rr range from 1 (perfect correlation) to −1 (perfect exclusion). Thus, a value close to 1 indicates reliable colocalization.

### Electron Micrograph

Retinal degeneration was examined with electron microscopy using retinal tissue sections. Fly eyes were prepared for electron microscopy using procedures described by Washburrn and O'Tousa (1992). Electron microscopy sections were ∼80–100 nm thick, stained first in 5% uranyl acetate in 50% EtOH and then in Reynold's lead citrate. The Hitachi H600 electron microscope was used to take electron micrographs. The micrographs shown in all figures are taken from ommatidia cross-sectioned at a depth of R1-R6 photoreceptor nuclei to present a similar view of various genotypes.

### Western Blot Analysis

Usually, 2–5 fly heads were homogenized in buffer A (20 mM Tris-HCl(pH 7.5), 100 mM NaCl, 5 mM MgCl2, 10% sucrose, 1% glycerol, 1 mM EDTA, 1% CHAPS and Complete Protease Inhibitor Cocktail) with pellet pestle. The homogenate was centrifuged at 4°C and 14,000 rpm for 2 min. The supernatant was separated on 12% SDS polyacrylamide gel and then transferred to PVDF membrane at 100 V for 1 hour. The membrane was blocked by 5% nonfat milk in TBST (TBS with 0.5% Tween 20) for 60 min. After blocking, the membrane was incubated with the mouse anti-rhodopsin antibody 4C5 (diluted 1∶5000, Developmental Studies Hybridoma Bank) for 60 min at room temperature or overnight at 4°C. The polyclonal rabbit anti-arrestin2 antibody (Genscript, NY) is diluted 1∶30,000. The anti-mouse or rabbit IgG HRP conjugated antibody was diluted 1∶5000 in TBST containing 5% skin milk and the membrane was washed with TBST for 30 min. The blotted membrane was detected with a homemade ECL solution for 1 min, and then exposed to X-ray film. The detected bands were quantified using the Quantity One (Bio-Rad).

### Time Course Analysis of Rhodopsin Endocytosis and Degradation

Newly eclosed *norpA^P24^* and *norpA^P24^*; *Osi21-RNAi* flies with UAS::Rh1-RFP under control of hs::Gal4 were kept in complete darkness for 24 h and then subjected to heat-shock for one hour in the 37°C incubator three times at six-hour interval. These flies were kept in the 25°C incubator for another day and then moved under 2900 lux light. Whole mount photoreceptors were examined by confocal microscopy at 24 h intervals for 96 h. At each time point, approximately 30 photoreceptor cells from at least five individuals were scored and categorized as follows: Type I (most Rh1-RFP localizes in the rhabdomere), Type II (Rh1-RFP localizes equally in the rhabdomere and the cytoplasm), Type III (most Rh1-RFP localizes in the cytoplasm) and Type IV (most Rh1-RFP disappears due to degradation). The Kolmogorov-Smirnov test for equality distribution was performed using the STATA software package.

## Supporting Information

Figure S1 **Identification of the mutation responsible for the** ***die4*** **phenotype.** (A) Genes deleted in the EXEL6028 genomic deficiency: 11 genes deleted in the Exel6028 genomic deficiency were identified based on Release 5.1 of the *Drosophila* genome. To identify a gene responsible for the *die4* phenotype, a complementation test was performed using existing fly mutant stocks. Only the *Osi21* loss-of-function mutant uncomplemented the *die4* phenotype. (B) Sequencing strategy: The *die4* chromosome is homozygous lethal. Therefore, the GFP balancer was used to distinguish the homozygous *die4* animal from the balanced heterozygotes. Fluorescently labeling the animal indicates the animal has the GFP balancer. Non-fluorescent homozygous *die4* embryos were selected for sequencing analysis. From sequencing analysis, three significant amino acid changes were recovered within *die4* (G149S, M181T, and F229L). (Arrow) *die4* homozygotes, (Arrowheads) GFP-balanced *die4*.
(TIF)

Figure S2 **Effect of** ***die4*** **on** ***rdgC^306^*** **- and** ***trp^1^*** **-dependent retinal degeneration.** (A–B) Newly eclosed flies were exposed to constant light (2900 lux) for four days. Maintenance of deep pseudopupil was examined by light microscopy. While the *rdgC^306^* fly lost its deep pseudopupil completely, the *die4; rdgC^306^* double mutant fly maintained its deep pseudopupil. (A) *rdgC^306^*, (B) *die4; rdgC^306^*. (C–D) Newly eclosed flies were exposed to constant light (2900 lux) for 7 days. Maintenance of deep pseudopupil was examined using light microscopy. While the *trp^1^* fly lost its deep pseudopupil completely, the *die4; trp^1^* double mutant fly maintained its deep pseudopupil. (C) *trp^1^*, (D) *die4; trp^1^*.
(TIF)

Figure S3 **Fractional shift of Rab7-positive vesicles in Iodixanol density gradients.** (A–B) Flies reared in complete darkness were exposed to bright light for 90 min. 30 fly heads were lysed and fractionated using a continuous Optiprep density gradients. Specific fractional shift of the Rab7-positive vesicles in the Rh1-positive franctions of *norpA^p24^; Rh1::Gal4* flies were compared to *norpA^p24^; Rh1::Gal4; UAS::Osi21-RNAi/+* in Western blot. (A) *norpA^p24^; Rh1::Gal4*, (B) *norpA^p24^; Rh1::Gal4; UAS::Osi21-RNAi/+*. (C) Quantification of relative intensity of the Rab7-positive vesicles in the Rh1-positive franctions of *norpA^p24^; Rh1::Gal4* flies were compared to that of *norpA^p24^; Rh1::Gal4; UAS::Osi21-RNAi/+*.
(TIF)

Figure S4 **Level of Rab5 and Rab7 expression.** (A) Western blot analysis was performed to check the level of Rab5 and Rab7 expression in the fly head sample. Note that YFP-Rab proteins are ectopically expressed only in the retina. *norpA^p24^; Rh1::Gal4, UAS:: YFP-Rab7/+* (lane 1), *norpA^p24^; Rh1::Gal4, UAS:: YFP-Rab5/+* (lane 2), *norpA^p24^; Rh1::Gal4, UAS:: GFP-Rab6/+* (lane 3). (B) Ectopically expressed YFP-Rab proteins and endogeneouls Rab proteins were quantified from the band intensity of triplicated immunoblots. The expression level of YFP-tagged Rab proteins was comparable to that of endogeneous Rab proteins, indicating overexpression of YFP-Rab proteins in the fly retina. Data are shown as the SEM.
(TIF)

Table S1 **Genomic deficiencies for deficiency mapping.** Note: *Df(2L)Exel8005* failed to cover the lethality in the *die4* chromosome. However, *Df(2L)Exel6028* failed to cover the suppressive phenotype of *die4*, indicating lethality caused by a gene in 22B2 to B8 region is not responsible for the suppressive phenotype of *die4*. (Y) complement, (L) lethal, (N) not complement.
(XLSX)

Table S2 **Complementation tests of** ***die4*** **with existing mutant alleles.** Note: Seven mutant alleles were tested. All except the loss-of-function mutant of *Osi21* complemented the *die4* in that the deep pseudopupil was lost within five days of constant light exposure.
(XLSX)

Table S3 **PCR Primers.**
(XLSX)

Text S1 **Method for Iodixanol density gradient analysis.**
(DOCX)
