# Supplementary figures and images for: Negative Regulation of the Novel norpAP24 Suppressor, diehard4, in the Endo-lysosomal Trafficking Underlies Photoreceptor Cell Degeneration
Source: PLoS Genet. 2013 Jun 6;9(6):e1003559. doi: 10.1371/journal.pgen.1003559 (PMC3674991; doi:10.1371/journal.pgen.1003559)

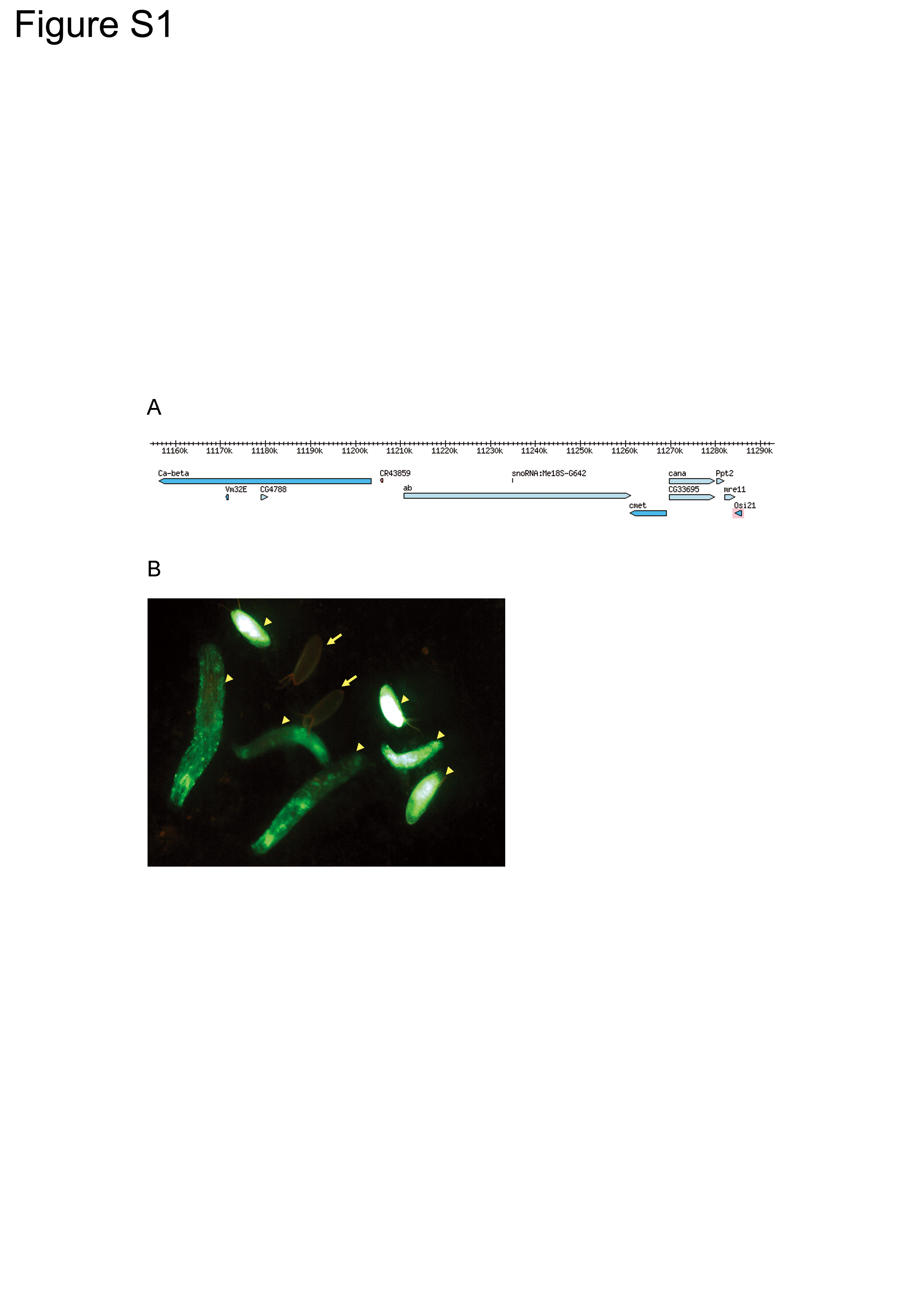

Supplement: Figure S1 — Identification of the mutation responsible for the die4 phenotype. (A) Genes deleted in the EXEL6028 genomic deficiency: 11 genes deleted in the Exel6028 genomic deficiency were identified based on Release 5.1 of the Drosophila genome. To identify a gene responsible for the die4 phenotype, a complementation test was performed using existing fly mutant stocks. Only the Osi21 loss-of-function mutant uncomplemented the die4 phenotype. (B) Sequencing strategy: The die4 chromosome is homozygous lethal. Therefore, the GFP balancer was used to distinguish the homozygous die4 animal from the balanced heterozygotes. Fluorescently labeling the animal indicates the animal has the GFP balancer. Non-fluorescent homozygous die4 embryos were selected for sequencing analysis. From sequencing analysis, three significant amino acid changes were recovered within die4 (G149S, M181T, and F229L). (Arrow) die4 homozygotes, (Arrowheads) GFP-balanced die4. (TIF) [file pgen.1003559.s001.tif]

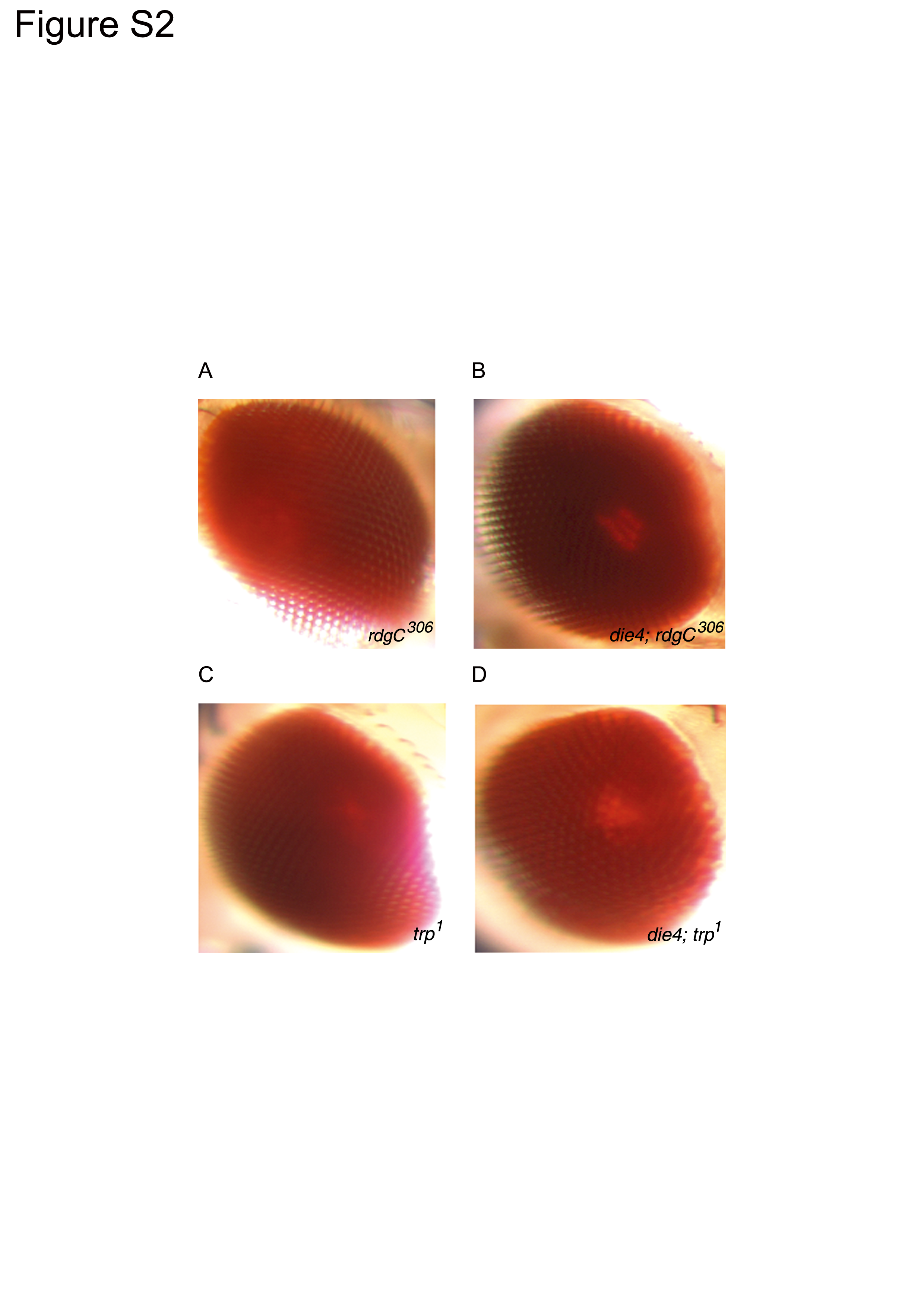

Supplement: Figure S2 — Effect of die4 on rdgC306 - and trp1 -dependent retinal degeneration. (A–B) Newly eclosed flies were exposed to constant light (2900 lux) for four days. Maintenance of deep pseudopupil was examined by light microscopy. While the rdgC306 fly lost its deep pseudopupil completely, the die4; rdgC306 double mutant fly maintained its deep pseudopupil. (A) rdgC306, (B) die4; rdgC306. (C–D) Newly eclosed flies were exposed to constant light (2900 lux) for 7 days. Maintenance of deep pseudopupil was examined using light microscopy. While the trp1 fly lost its deep pseudopupil completely, the die4; trp1 double mutant fly maintained its deep pseudopupil. (C) trp1, (D) die4; trp1. (TIF) [file pgen.1003559.s002.tif]

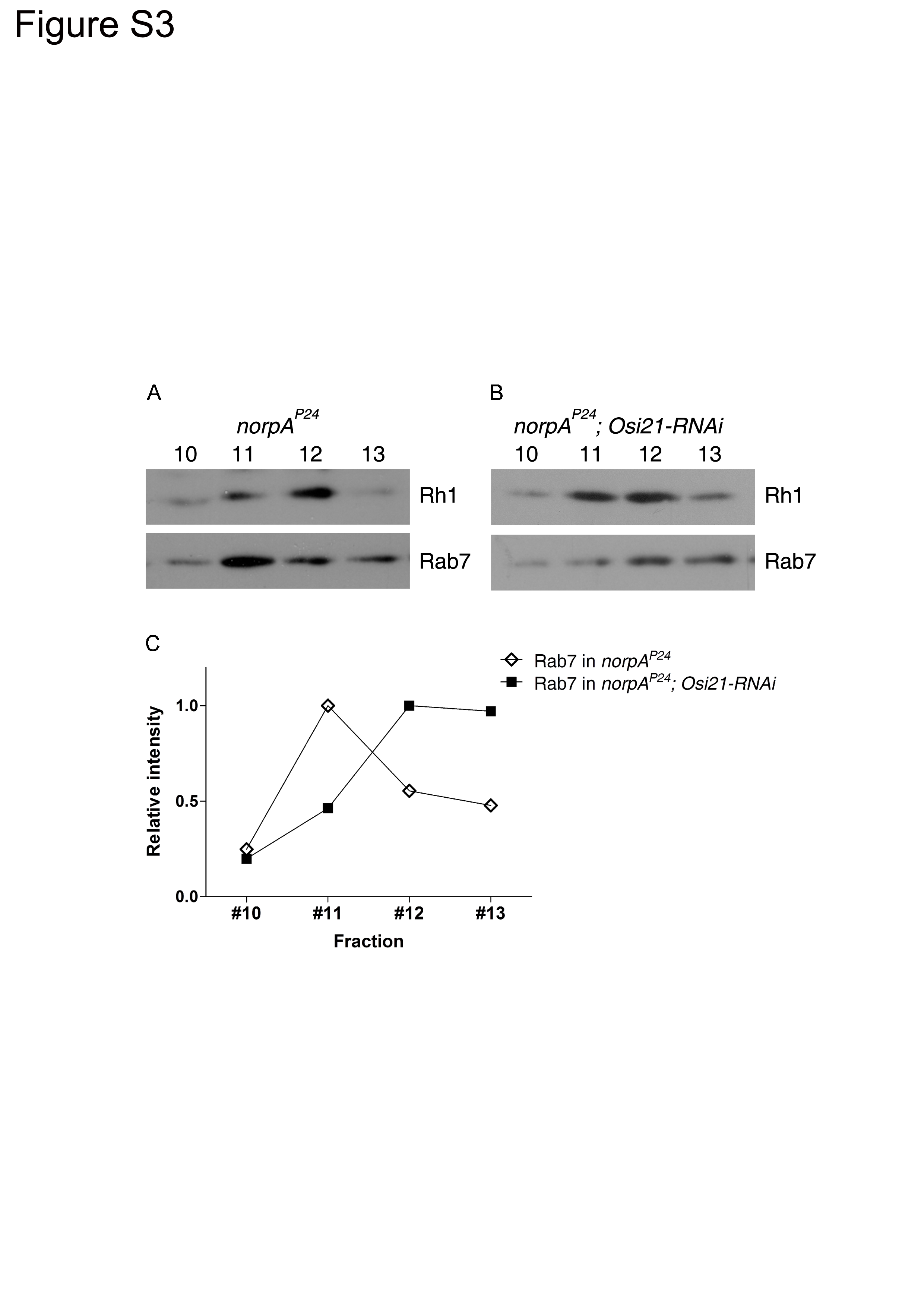

Supplement: Figure S3 — Fractional shift of Rab7-positive vesicles in Iodixanol density gradients. (A–B) Flies reared in complete darkness were exposed to bright light for 90 min. 30 fly heads were lysed and fractionated using a continuous Optiprep density gradients. Specific fractional shift of the Rab7-positive vesicles in the Rh1-positive franctions of norpAp24; Rh1::Gal4 flies were compared to norpAp24; Rh1::Gal4; UAS::Osi21-RNAi/+ in Western blot. (A) norpAp24; Rh1::Gal4, (B) norpAp24; Rh1::Gal4; UAS::Osi21-RNAi/+. (C) Quantification of relative intensity of the Rab7-positive vesicles in the Rh1-positive franctions of norpAp24; Rh1::Gal4 flies were compared to that of norpAp24; Rh1::Gal4; UAS::Osi21-RNAi/+. (TIF) [file pgen.1003559.s003.tif]

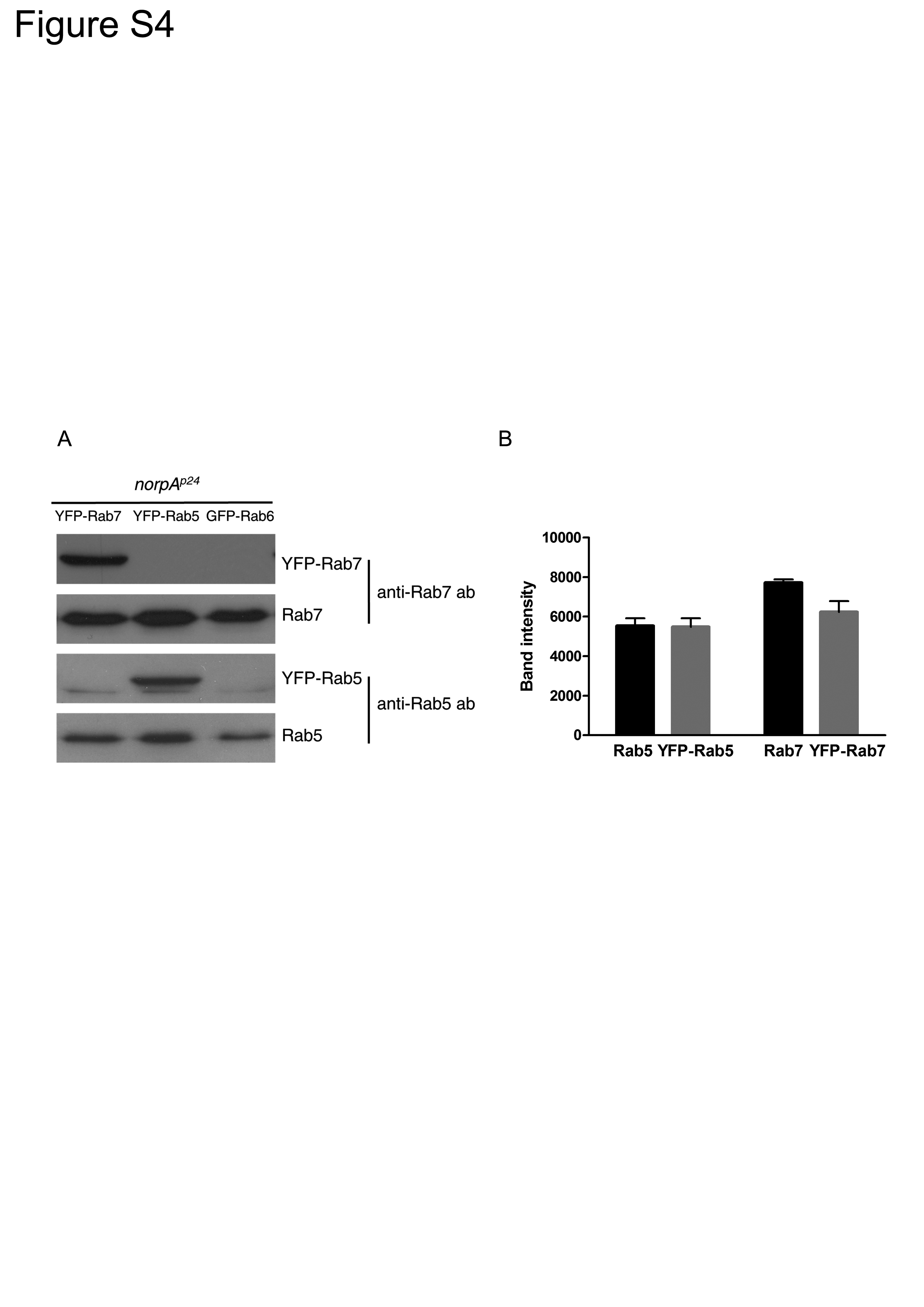

Supplement: Figure S4 — Level of Rab5 and Rab7 expression. (A) Western blot analysis was performed to check the level of Rab5 and Rab7 expression in the fly head sample. Note that YFP-Rab proteins are ectopically expressed only in the retina. norpAp24; Rh1::Gal4, UAS:: YFP-Rab7/+ (lane 1), norpAp24; Rh1::Gal4, UAS:: YFP-Rab5/+ (lane 2), norpAp24; Rh1::Gal4, UAS:: GFP-Rab6/+ (lane 3). (B) Ectopically expressed YFP-Rab proteins and endogeneouls Rab proteins were quantified from the band intensity of triplicated immunoblots. The expression level of YFP-tagged Rab proteins was comparable to that of endogeneous Rab proteins, indicating overexpression of YFP-Rab proteins in the fly retina. Data are shown as the SEM. (TIF) [file pgen.1003559.s004.tif]
