# Supplementary material for: Negative Regulation of the Novel norpAP24 Suppressor, diehard4, in the Endo-lysosomal Trafficking Underlies Photoreceptor Cell Degeneration
Source: PLoS Genet. 2013 Jun 6;9(6):e1003559. doi: 10.1371/journal.pgen.1003559 (PMC3674991; doi:10.1371/journal.pgen.1003559)
Supplement: Text S1 — Method for Iodixanol density gradient analysis. (DOCX) [file pgen.1003559.s008.docx]

**Supplementary Methods**

**Iodixanol density gradient analysis**

Flies reared in complete darkness were exposed to bright light for 90 min. 30 fly heads were lysed in cell fractionation lysis buffer (10 mM Hepes(pH 7.5), 1mM EDTA, 250 mM sucrose, 20% Optiprep^TM^ (SIGMA;D1556), complete mini EDTA free protease inhibitor (Rosche)) using a plastic pestle. The whole lysates were centrifuged at 4 °C, 3000xg for 10 min. Supernatants were loaded on 20% Optiprep^TM^ buffer(10 mM Hepes(pH 7.5), 1mM EDTA, 250 mM sucrose, 20% Optiprep^TM^) and were centrifuged at 15°C, 363,000g for 3 h using a NVT-90 rotor (Beckman). The Fraction was harvested from the bottom of the puncture with needle. Each fraction was analyzed in SDS-PAGE and immunoblot.
